# False Memories of Emotional and Neutral Words

**DOI:** 10.1155/2008/587239

**Published:** 2008-04-11

**Authors:** Jennifer El Sharkawy, Katarina Groth, Céline Vetter, Anna Beraldi, Kristina Fast

**Affiliations:** ^1^Neuro-Cognitive PsychologyDepartment PsychologyLudwig-Maximilians-Universität MünchenGermany; ^2^Division of Clinical Psychology and PsychophysiologyDepartment of PsychiatryLudwig-Maximilians-Universität MünchenGermany

**Keywords:** False memory, emotion, valence, Deese-Roediger-McDermott paradigm

## Abstract

This study used the Deese-Roediger-McDermott paradigm [34] to investigate the direction and the extent to which emotional valence in semantic word lists influences the formation of false memories (FM). The experimental paradigm consisted of 1) a study phase (learning of neutral and negative lists of words semantically associated to a non-presented critical lure (CL), 2) a free recall phase, and 3) a recognition phase. Participants had to indicate whether the displayed item was "new" (new item or non-studied CL) or “old” (studied list item). CL associated with negative word lists elicited significantly more FM than CL associated with neutral word lists. This finding is in contrast to previous work showing that emotional words elicit fewer FM than neutral words. The results of our study also suggest that valence is capable of influencing emotional memory in terms of encoding and retrieval processes.

## 1. Introduction

It has been well established that memory for emotional events is different than for neutral events [2, 12,15–17,24]. These studies could all show that distinct neurobiological regions underlie memory of neutral and emotional content[5]. There is emerging evidence that not only neutral and emotional memory can be differentiated, but also that positive and negative content are subjected to distinct processing mechanisms. The valence hypothesis [7,8] posits distinct neural underpinnings for the processing of emotional valent and neutral stimuli which is supported by recent neuroimaging studies (e.g. [13]). It is generally assumed that valence is a valid criterion to study memory processes [23,25].

The Deese-Roediger-McDermott (DRM) paradigm [9,10,34] is widely used to study the phenomenon of false memories. Participants are typically presented with word lists highly associated to a non-presented word, the critical lure (CL). In subsequent recognition phases the CL is often mis-categorized as a presented word. Such events are referred to as false memories (FM). The DRM paradigm has been used to examine memory systems, including memory of language representations [4,5,40], memory effects of aging [11,14, 22] and memory in clinical populations [27].

Pesta and colleagues [32] investigated whether CL derived from emotional content would be more distinct than neutral CL, and therefore less likely to be

[1]Contributed equally and are jointly considered first author.
[2]Current address: University Clinic for Children and Adolescent Psychiatry, Psychotherapy and Psychosomatic, Ludwig-Maximilians-University, Munich, Germany.
[3]Current address: Transfer Centre for Neuroscience and Learning, Institute for Neurosciences and Learning, University Hospital Ulm, Germany.
[4]Current address: Psychology Department of the University of Warwick, UK. Institute of Medical Psychology, Ludwig-Maximilians-University, Munich, Germany.
[*]Corresponding author: Dr. Kristina Fast, Department of Psychiatry, Division of Clinical Psychology and Psychophysiology, Section of Neurocognition, Ludwig-Maximilians-University, Munich, Nußbaumstr. 7, D- 80336 München. Tel.: +49 89 5160 5565; Fax: +49 89 5160 5562; E-mail: kristina.fast@med.uni-muenchen.de.
[5]The term emotional content refers to stimuli that have been rated on a scale of arousal ("calm – exciting"), or on a scale of emotional valence ("pleasant – unpleasant") or a combination of both.

ISSN 0953-4180/08/$17.00 © 2008 – IOS Press and the authors. All rights reserved

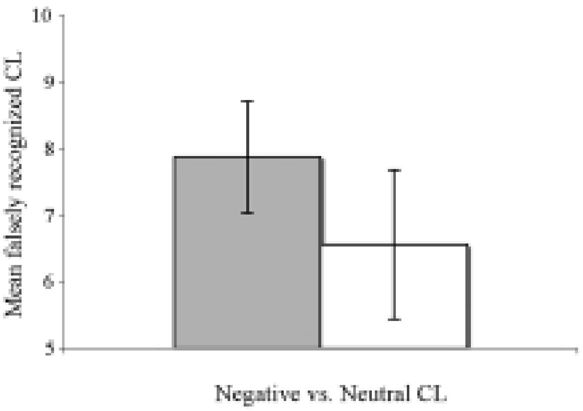

Fig. 1. This graph visualizes the mean number of falsely recognized words for negative and neutral non-presented associates (CL) during recognition phase. Subjects produced significantly more false alarms when the non-presented associate was a negative word.

falsely recognized. They found enhanced memory for emotional CL that were phonologically associated to lists of neutral, orthographically similar words in comparison to neutral CL. Importantly, the authors employed not only emotionally laden stimuli, but also taboo words (e.g. whore), which leads to enhanced recognition/rejection capabilities [38]. Thus, differential retrieval heuristics may be employed that would reduce false recall/recognition [19,36].

The present study aims to investigate how semantically associated words, either neutral or of negative emotional valence, modulate the production of FM. Adolphs and Damasio [1] postulated that emotional situations were paramount to survival. Therefore, semantically relevant emotional content could create stronger inter-item associations. In turn, this would result in enhanced FM production, a prediction consistent with the Activation Monitoring Framework (a review is beyond the scope of this article, but see [33,35]).

## 2. Method

### *2.1. Participants*

32 healthy volunteers (16 female) with a mean age of 26.2 years (sd = 3.3 yrs) participated in the study. All were native German speakers with university education. Mean verbal IQ, assessed with the MWT-B [26], was 116.25 (sd = 11.94).

### *2.2. Material*

28 lists of converging associates were used, each consisting of 12 nouns (to avoid interference due to distinct neural processing of different word categories [6,31, 37]) strongly associated to a CL. All word lists were taken from the "Uni-Leipzig-database" [39]. In the case that insufficient nouns were supplied by the database (<5%), backward association lists were created via an independent sample ($n = 18$). Previously, this has been shown to be a valid approach [3]. To sacrifice the semantic associative strength within the word lists, we included highly associated words despite differential word length, word frequency or distinct levels of word concreteness. These factors will be considered in the results section. Word concreteness was evaluated by a further independent sample ($n = 12$) who rated all words on a Likert scale from one to five (1 = highly concrete, 5 = highly abstract).

### *2.3. Procedure*

The experiment consisted of a study phase, a free recall phase and a recognition phase (all proceeding in a subsequent manner) followed by the assessment of verbal IQ. During the study phase, all 28 negative and neutral word lists were presented on a computer screen for 15 sec in a pseudo-randomized order. Sets were counter-balanced across participants. CL were not included in this phase. Participants were instructed to learn all words for a subsequent memory test. In the recall phase participants were asked to verbally list the remembered items (max. 5 min) and experimenters recorded their responses. In the recognition phase, 60 words (20 old list items, 20 CL and 20 unrelated words, each 50% neutral, 50% negative and pseudo-randomly selected) were successively presented (until response or max. 5 sec) via monitor and participants indicated whether the word was "old" (studied list item) or "new" (new unrelated word or CL). Responses were instructed to be given as spontaneously and as accurately as possible via mouse button press. The experiment lasted approximately 30 min. Each participant completed all 3 phases.

## 3. Results

Two tailed t-tests for paired samples (alpha level 0.05) were calculated for free recall, recognition performance, and FM occurrence. The dependent variable

Table 1
Descriptive Statistics: Word-Recognition

| | Negative | | | Neutral | | |
|---|---|---|---|---|---|---|
| | Old | CL | Intrusion | Old | CL | Intrusion |
| Mean | 5.47 | 7.88 | 1.34 | 5.31 | 6.56 | 0.97 |
| SD | 1.92 | 1.68 | 1.33 | 1.15 | 2.24 | 1.15 |
| correct | | 16.25 | | | 17.84 | |

Note: Old = previously learned list item, CL = associated non-presented item, Intrusion = non-associated new item, SD = standard deviation.

was "response type" (correct = correctly recognized old or new list item; incorrect = false memory [CL], intrusions [new items] or failed recognition [old items]), the independent variable was "list type" (neutral versus negative CL-list).

### *3.1. Recall*

Participants correctly remembered an average of 49.28 words. A comparison between the mean number of negative (mean = 21.31, sd = 8.15) and neutral words (mean = 22.75, sd = 10.23) recalled was non-significant, [$t(31) = -0.706$, $p = 0.486$]. The mean number of negative and neutral CL in free recall (means = 1.81 and 2.16, sd = 1.35 and 1.9, respectively) did not differ significantly, [$t(31) = 1.046$, $p = 0.304$]. Participants were equally prone to intrusions, independent of negative (mean = 0.9, sd = 1.16) or neutral valence (mean = 0.77, sd = 1), [$t(31) = 0.436$, $p = 0.666$].

### *3.2. Recognition*

The descriptive statistics for recognition are depicted in Table 1. Mean number of correct responses [$t(31) = 0.524$, $p = 0.604$] and the mean number of intrusions [$t(31) = 1.506$, $p = 0.142$] did not differ significantly between neutral and negative words. Negative CL were significantly more often falsely recognized than neutral CL [$t(31) = 2.860$, $p < 0.01$]. Finally, the mean total correct recognition was significantly better for neutral words [$t(31) = -2.597$, $p < 0.05$]. Cohen's d' statistic for dependent samples was calculated to evaluate the effect size ($d' = 0.66$). Emotional valence thus appears to have a relatively large effect on false memory production. Figure 1 visualizes this main result.

The proportion of falsely recognized CL outnumbered the correct recognition of list items in the negative [$t(31) = -5.445$, $p < 0.001$] and neutral [$t(31) = -2.968$, $p < 0.01$] conditions. To exclude the possibility that subjects performed at chance level and to assure that the lists had been learned, mean list item recognition was compared to the mean number of intrusions. The proportion of neutral list items recognized was significantly higher than for neutral intrusions [$t(31) = 17.778$, $p < 0.001$]. The same was observed for negative list items [$t(31) = 12.416$, $p < 0.001$] when compared to the number of negative intrusions.

Additionally, the factors word length, word frequency and level of concreteness were analysed. Word length of negative (mean = 1.79 syllables, sd = 0.96) and neutral CL (mean = 1.5, sd = 0.65) was comparable [$t(13) = 1.000$, $p = 0.336$]. However, negative list items (mean = 2.42 syllables, sd = 0.84) were significantly longer than neutral ones (mean = 1.94, sd = 1.03), [$t(177) = 4.801$, $p < 0.000$]. Similarly, word concreteness for negative CL (mean = 2.30, sd = 0.78) and negative list items (mean = 2.27, sd = 0.91) was rated more abstract than for neutral CL (mean = 1.37, sd = 0.40), [$t(13) = 4.904$, $p < 0.000$] and neutral list items (mean = 1.48, sd = 0.53), [$t(177) = 10.774$, $p < 0.000$]. Word frequency of negative (mean = 17865.21, sd = 22765.29) and neutral CL (mean = 29937.93, sd = 61857.37) as well as of negative (mean = 16334.77, sd = 27919.92) and neutral list items (mean = 21452.85, sd = 55965.46) was comparable [$t(13) = 0.661$, $p = 0.520$; $t(177) = 1.113$, $p = 0.267$; respectively].

## 4. Discussion

The main goal of this study was to compare the FM rates elicited from lists of words semantically associated with a negative or a neutral CL. We found that negative CL were more often falsely recognized, which is in contrast to previous work done in this field [20, 21,23,29,32] but in line with work done by Adolphs and Damasio [1] as well as theories like the Activation Monitoring Framework [33,35]. The results revealed that both negative and neutral CL were recognized more often than negative and neutral list items (a common finding, e.g. [30]) and that the correct recognition of list items was greater for old than for new items. These findings show that the word lists were successful in eliciting FM and that subjects did learn the lists.

With regard to the influence of word length in FM occurrence, negative words were significantly longer in this experiment than neutral items. Word length is commonly thought to increase word distinctiveness [35] and thus, one would expect lower frequency of FM. However, our results followed an opposite pattern. One might conclude that word length is less important in influencing FM occurrence than semantic associative strength and emotional valence.

Similarly, negative items were judged as being more abstract than neutral items. Previous studies [18,28] report higher false recognition rates with increasing levels of abstractness. This is in line with our results. It remains unclear whether negative words trigger more FM due to higher levels of abstractness or due to their emotional value. Therefore, this abstractness question deserves further attention in context with the DRM paradigm.

In conclusion, our results highlight a novel twist on a widely used memory paradigm. Additionally, the results are theoretically supported by the activation monitoring framework in that emotional content of words might also increase inter-item associations and thus decrease memory precision. Finally, our results raise numerous possibilities for future research endeavours.
